# 10 Fr S-Type Plastic Pancreatic Stents in Chronic Pancreatitis Are Effective for the Treatment of Pancreatic Duct Strictures and Pancreatic Stones

**DOI:** 10.1155/2018/6056379

**Published:** 2018-10-25

**Authors:** Ken Ito, Naoki Okano, Seiichi Hara, Kensuke Takuma, Kensuke Yoshimoto, Susumu Iwasaki, Yui Kishimoto, Yoshinori Igarashi

**Affiliations:** Division of Gastroenterology and Hepatology, Toho University Omori Medical Center, Tokyo, Japan

## Abstract

**Aim:**

Endoscopic pancreatic stenting for refractory pancreatic duct strictures associated with impacted pancreatic stones in chronic pancreatitis cases has yielded conflicting results. We retrospectively evaluated the efficacy of endoscopic treatment in chronic pancreatitis patients with pancreatic duct strictures.

**Methods:**

Pancreatic sphincterotomy, dilatation procedures, pancreatic brush cytology, and pancreatic juice cytology were routinely performed, and malignant diseases were excluded. After gradual dilatation, a 10 Fr plastic pancreatic stent was inserted. The stents were replaced every 3 months and removed after the strictures were dilated. Statistical analyses were performed to determine the risk of main pancreatic duct restenosis.

**Results:**

Endoscopic pancreatic stents were successfully placed in 41 of a total of 59 patients (69.5%). The median duration of pancreatic stenting was 276 days. Pain relief was obtained in 37 of 41 patients (90.2%). Seventeen patients (41.5%) had recurrence of main pancreatic duct stricture, and restenting was performed in 16 patients (average placement period 260 days). During the follow-up period, pancreatic cancer developed in three patients (5.1%). Multivariate analysis revealed that the presence of remnant stones after stenting treatment was significantly associated with a higher rate of main pancreatic duct restenosis (*p* = 0.03).

**Conclusion:**

The use of 10 Fr S-type plastic pancreatic stents with routine exchange was effective for both short-term and long-term outcomes in chronic pancreatitis patients with benign pancreatic duct strictures and impacted pancreatic stones.

## 1. Introduction

Chronic pancreatitis is a progressive, irreversible inflammatory disease characterized by pain, which is the symptom that requires treatment in most cases [1]. This disease is thought to be caused by increased pressure within the pancreatic ductal system and/or pancreatic parenchyma, secondary to the outflow obstruction of the main pancreatic duct (MPD) [2].

It has been reported that endoscopic pancreatic duct stenting provides both short-term and long-term relief from persistent or relapsing pain in severe chronic pancreatitis with distal ductal strictures and proximal dilation [3–8].

Several stents of various shapes and diameter have been used for endoscopic pancreatic stenting (EPS) [4–7, 9–14]. In consideration of the migration of the pancreatic stent, a polyethylene straight-type PS (Amsterdam type) [5], with 1 cm interval side holes, were the common PS for endoscopic pancreatic stenting [5]. We had an experience of using Amsterdam-type PS with a case of back pain from the early stage, in which we were forced to remove and exchange in the early timing. So, we started and preferred to use a polyolefin elastomer material with double-bended type (S shape) [15–17], which was a more soft material and suitable at the main pancreatic duct. This is the first reason we only use S-type pancreatic stent in our Hospital.

In addition, endoscopic pancreatic stenting in Japan has been approved for medical health insurance coverage in April 2012, and at that time, only S-type plastic pancreatic stent (Olympus Co.) was the only plastic stent which was funded by the national medical insurance in Japan. From these two reasons, we evaluated the efficacy of approved medical health insurance coverage pancreatic stents. The European Society of Gastrointestinal Endoscopy (ESGE) Clinical Guideline recommended the use of 10 Fr diameter plastic stents in chronic pancreatitis associated with severe strictures [18]. S-type plastic stents have proven to be safe and efficient for the treatment of pancreatic duct strictures by EPS [12, 13, 19, 20]. MPD obstruction has been reported to be caused by strictures (47%), stones (18%), or a combination of both (32%) in most patients [4, 6, 7, 10, 13, 21]. The combination of extracorporeal shock wave lithotripsy (ESWL) and EPS is considered to be the treatment modality for ameliorating pain in patients with chronic pancreatitis [4, 7, 17, 22–29]. However, only a few cases of severe pancreatic duct strictures with impacted pancreatic stones in patients using 10 Fr S-type plastic pancreatic stents (plastic PS) have been reported so far. The present study retrospectively evaluated the short-term and long-term efficacies and outcomes of using 10 Fr S-type plastic PS for the treatment of pancreatic duct strictures and impacted pancreatic stones in patients with chronic pancreatitis.

## 2. Methods

### 2.1. Patients

From May 2005 to November 2013, 148 chronic pancreatitis and pancreatolithiasis patients were treated by endoscopic stone extraction and ESWL at Toho University Omori Medical Center, Tokyo, Japan. Among them, 59 patients, who underwent 10 Fr S-type pancreatic stent placement and were followed up for over 12 months, were selected for evaluation in the present study.

Adaptation for EPS was based on clinical symptoms (e.g., abdominal pain), presence of pancreatic duct stones in the Santorini or Wirsung ducts, detection of upstream MPD dilatation by diagnostic imaging (ultrasonography, contrast enhanced computed tomography (CT), and magnetic resonance cholangiopancreatography), and the presence or absence of abdominal complaints with exacerbation of glucose tolerance and diabetic mellitus.

EPS was not funded by the national medical insurance of Japan until April 2012; therefore, this study was conducted with the approval of the Toho University Omori Medical Center's Institutional Review Board and in accordance with the Declaration of Helsinki. Clinicopathological data were obtained from patients' medical records. Written informed consent was obtained from each patient before the procedures.

### 2.2. EPS Equipment and Procedures

All procedures were performed with a TJF240 or TJF260V duodenoscope (Olympus Co., Tokyo, Japan). Endoscopic pancreatic sphincterotomy (EPST) has consistently been performed before MPD stenting [21]. When selective MPD cannulation was difficult, precutting was performed with EPST as a secondary procedure [30]. After identification of the pancreatic duct stricture via pancreatography, a guidewire was negotiated through its tail, as close as possible to the MPD, and dilatation was attempted.

**Figure 1:**
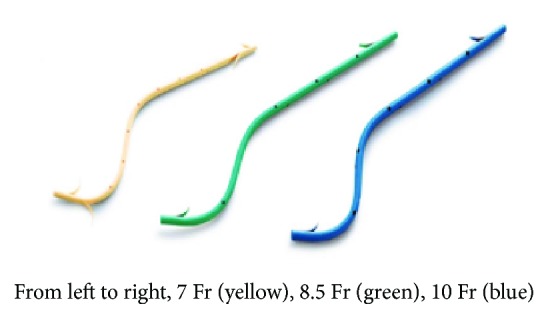
Devices of pancreatic stents.

Routine pancreatic cytology was performed before commencing with the dilatation procedure to confirm the absence of malignancy in the MPD stricture. Although we typically used 0.035-inch Revowave standard-type and Revowave hard-type guidewires (Piolax Medical Devices Inc., Kanagawa, Japan), a 0.025-inch VisiGlide or VisiGlide 2 guidewire (Olympus Co.) was also used in patients with severe strictures. Similarly, despite the use of a dilation catheter (SBDC; Cook Co., Winston-Salem, NC, USA) or a 6 mm diameter balloon catheter for endoscopic pancreatic duct dilation (EPDBD: MaxPass; Olympus Co.) for stricture dilation before stenting, a Soehendra stent retriever (SSR; Cook Co.) was used as an alternative device to dilate the more challenging strictures [31–33]. Pancreatic duct stones have been effectively treated by a combination therapy of both EL and ESWL as a first-line treatment method [34]. ESWL was first started with an electromagnetic lithotripter (Lithoskop; Siemens AG, Munich, Germany); a wire-guided basket (FG-V436P Tetra-V wire-guided basket; Olympus Co.) was then introduced after ESWL fragmentation of the ductal stones. In instances where ESWL was unsuccessful, electrohydraulic lithotripsy (EHL) was performed as a second attempt using the 10 Fr SpyGlass Direct Visualization system (Boston Scientific, Natick, MA). An S-type plastic PS (Olympus Co.) was used for the MPD stricture (Figure 1). A pancreatic stent of adequate diameter (7 or 8.5 Fr) and length (4, 6, or 8 cm) was used during stone fragmentation. In cases where pancreatic stenting was unsuccessful owing to large stone burden, a 5 Fr ENPD (Cook Co.) was temporarily placed until fragmentation had occurred. After the residual stones were almost crushed by ESWL, they were removed endoscopically, and a 10 Fr S-type plastic PS was finally inserted into the exposed MPD stricture. Follow-up data were collected after the placement of the 10 Fr stent. EPS exchange and pancreatic duct brush cytology were performed every 3 months during the duration of stent application. Additional stone extraction was performed in the presence of small stones that remained in the MPD.

**Figure 2:**
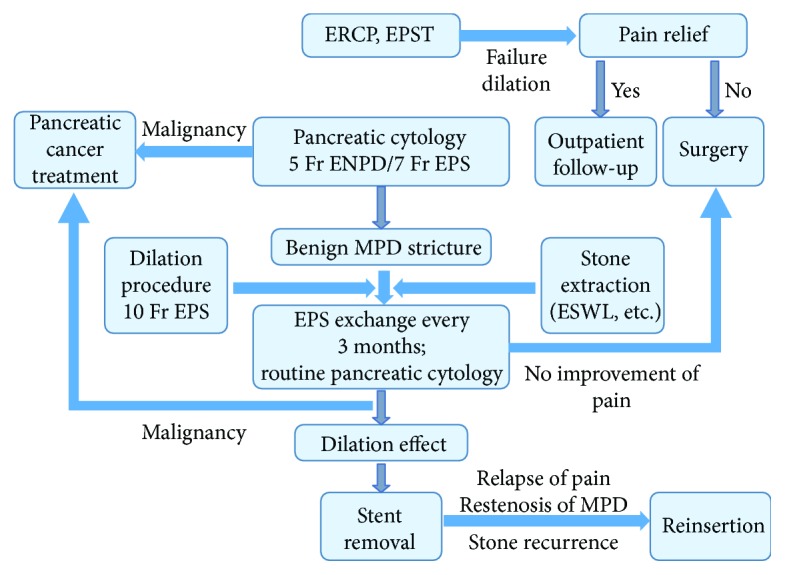
Algorithm of the treatments.

Finally, the dilation effect was revealed after repeated stent exchanges for at least 3 months to 1 year, wherein the pancreatic stent was removed and the patient was followed up in the outpatient department. Patients presenting with no improvement in pain symptoms after the stent-placement procedures were referred to the surgeon. In cases where malignancy was revealed by cytology, the stenting therapy was interrupted and appropriate treatment (surgery or chemotherapy) was initiated. Stent reinsertion was performed in patients with pain relapse, MPD restenosis, and stone recurrence after stent removal. These algorithms are shown in Figure 2.

### 2.3. Postprocedural Evaluation and Patient Follow-Up

Clinical outcomes were evaluated according to the following parameters: technical success of stent placement, number of stent exchanges, placement periods, effect of pain relief, adverse events, coexisting rates of malignant disease, and both restenosis as well as restenting rates. The risk factors for MPD restenosis were as follows: alcohol as an etiology of chronic pancreatitis, resumption of alcohol after stent removal, continued smoking habit, presence of single or multiple stones, retention of stones after stent removal, recurrence of stones during the stenting treatment, and stricture at the body of MPD or Santorini duct. In addition to these factors, re-stricture during stenting treatment, re-stricture with diffuse pancreatic stones, and the presence of re-strictures and diffuse stones due to alcohol consumption are also considered as risk factors for pancreatic cancer.

### 2.4. Definition of Events

The primary study outcome was pain relief (control) and dilation during both short-term and long-term evaluation of the clinical success. The secondary outcome was defined by the diagnosis of malignancy following cytology during stent exchange and restenosis after the stent-free term.

Short-term and long-term periods were set for each of the two groups, the stent-placement success group and the stent-placement failure group. For the success group, short-term was defined as the period when the first repeat EPS was placed, whereas long-term was defined as the period when the stent was removed after the first repeat stent exchange session. In the stent placement failure group, short-term was defined as the period during which the first admission attempting to place the EPS (actually, it only displays the clinical outcomes) was performed, whereas long-term was defined as the period after the admission term of the first failure attempt of the EPS placement.

### 2.5. Statistical Analysis

Statistical analysis was performed using SPSS for Windows, version 11.0J (SPSS Inc., Chicago, IL). Absolute numbers and percentages as well as median (with interquartile range) are computed to describe patients' age, stent-placement periods, number of stent exchanges, and follow-up periods. Categorical values were compared by chi-square test, and continuous variables were compared using Mann-Whitney *U* tests. Univariate logistic regression analysis was performed to identify risk factors associated with MPD restenosis and pancreas cancer. Factors with *p* < 0.05 were retained for multiple logistic regression analysis, and those demonstrating statistical significance (*p* < 0.05) on a multivariate analysis were considered verifiable predictive factors.

## 3. Results

### 3.1. Patient Characteristics

The characteristics of the 59 patients in this study are presented in Table 1. This study included 47 males and 12 females, with an age range of 25–81 years (median, 56 years). The etiology of chronic pancreatitis was alcohol abuse in 51 patients, idiopathic in seven, and iatrogenic in one patient. Severe strictures were located in the head (48), body (6), genu (3), and the Santorini duct (2) of the patients. All patients had pancreatic stones in the MPD (a single stone in 16 patients and multiple stones in 43 patients). There were 53 smokers and six nonsmokers.

### 3.2. Short-Term Outcomes during Plastic PS Placement

Table 2 summarizes the short-term outcomes during EPS placement. The stents were successfully placed in 41 of 59 patients (69.5%). The median duration of pancreatic stenting was 276 days (range, 30–589 days). In total, 169 pancreatic stents were placed during this study, and PPS placement was performed approximately 1–16 times (median, 4 times) during the stenting session. The median number of times endoscopic retrograde cholangiopancreatography (ERCP) was performed from the first ERCP until 10 Fr plastic PS placement was 3.5. Thirty-seven (90.2%) of 41 patients who received EPS placement achieved pain relief. However, 15 patients (83.3%) in the EPS-failure group also achieved pain relief indicating no difference when compared with the EPS placement group. Among the 18 patients without EPS placement, 10 followed ESWL, four underwent observation at the outpatient department, and four presented with continuing abdominal complaints requiring surgical treatment. The reasons for plastic PS placement failure in the 18 patients included inability to properly cannulate MPD with EPST (10 patients) and inadequate pancreatic stone lithotripsy (eight patients). However, successful stone extraction was obtained in four patients, whereas in 14 patients the extraction proved to be a failure revealing significant differences between the two groups. EPST or precut was performed in all patients. The precut technique was performed in four out of 41 patients (9.8%) in the EPS-success group, and in 15 of the 18 patients (83.3%) in the EPS-failure group. For MPD dilation, SSR was effective in 24 patients (58.5%) because of the presence of severe strictures. Stent-related complications occurred in seven (3.6%) patients. Plastic PS had to be removed in three patients because of continuing abdominal pain. Furthermore, three out of four stent-occlusion cases resulted in severe complications; one patient presented with pancreatic abscess, one with colon fistula, which was treated under observation, while the third patient presented with splenic abscess, which was subsequently treated by percutaneous drainage. All the three afore-mentioned patients had multiple diffuse stones in the tail of the MPD.

### 3.3. Long-Term Outcomes

Table 3 shows the long-term follow-up outcomes of the 59 patients. The median follow-up periods were 27 months after EPS insertion and 36 months in the EPS-failure group, indicating no differences between the two groups. Recurrence of MPD stricture was observed in 17 (41.5%) of the 41 patients. The median re-stricture time after removal of the first EPS was 191 (58–919) days. Re-stricture was observed in seven patients as a result of retention of MPD stones. Furthermore, exacerbation of chronic pancreatitis was noted because of resumption of alcohol in four patients and the recurrence of stones in two other patients. Sixteen patients (39.0%) received restenting (second placement), and the median period of these EPS placements was 260 (113–759) days. During this follow-up period, pancreatic cancer had developed in 3 (7.3%) patients, which was diagnosed 211 days after the first stent removal. Pancreatic duct cytology was performed in one patient after abdominal CT, whereas the two other patients were diagnosed by pancreatic duct cytology during routine stent exchange. One patient with pharyngeal cancer was diagnosed 1613 days after the first ERCP. Plastic PS placement had failed, but fortunately, pain relief was achieved after precut addition. After pain relief, upper esophagogastroduodenoscopy and abdominal CT were performed every year at the outpatient department.

### 3.4. Risk Factors for MPD Restenosis and Factors of Pancreas Cancer

Tables 4 and 5 show the risk factors for MPD restenosis. Among the seven risk factors revealed by univariate analysis, “remaining stones after stent removal” and “stricture at the body of the MPD” were found to be associated with MPD restenosis. In the multivariate analysis, “remaining stones after stent removal” was identified as an independent factor of MPD restenosis. No significant risk factors for pancreatic cancer were observed in this study (Table 6).

## 4. Discussion

In the present study, we retrospectively evaluated the usefulness and long-term outcomes of chronic pancreatitis with MPD strictures and pancreatic stones. 10 Fr S-type plastic PS were successfully placed in 69.5% of 59 patients in this study. The success rates of EPS placements have been reported to range from 85%–98% [4–6], which is higher than that observed in the present study (69.5%). However, contrary to previous reports [35], most patients in this study (11 of 14 patients with 10 Fr S-type plastic PS and stone extraction failure) presented with diffuse pancreatic stones. These findings suggest that the inclusion of patients with diffuse pancreatic stones along with MPD obstruction had a negative influence on the technical success and may be responsible for the low clinical success rates. Immediate pain relief was obtained in 37 of the 41 patients (90.2%) with 10 Fr S-type plastic PS placement, which is in agreement with previously published reports where the placement of stents has been reported to be followed immediately by pain relief in approximately 65%–95% patients [4–7, 10, 13, 14, 36]. As observed in the present study, it takes several sessions of ERCP to place a 10 Fr plastic PS in the duct. Impacted pancreatic stones (diffuse or large) or severe PD strictures inhibit deep pancreatic cannulation, and it is challenging to place a 10 Fr S-type plastic PS during the first session. However, it is important to place a small-diameter stent early in the session to decompress the dilated MPD [8]. Pain relief is expected to be achieved in the early session, after which stone fragmentation and removal of MPD obstruction are performed followed by the placement of the 10 Fr S-type plastic PS over several steps. Furthermore, it is important to traverse the MPD obstruction using several guidewires; stricture-dilation procedures using SSR have proven to be useful in previous studies [32, 33]. In the present study, SSR was utilized in 58.6% patients with MPD strictures, indicating its usefulness as one of the key facilitators in MPD dilatation.

In addition, this study shows that the EPST or precutting techniques used in the EPS failure cases were effective in relieving pain. In one of our previous reports, we have shown that MPD hypertension is decreased by using either one of these techniques, leading to a reduction in abdominal pain [34]. Placement of stents is a relatively easy, acceptable, safe, and effective procedure, which can be used to alleviate the symptoms of chronic pancreatitis rapidly.

On the other hand, complications including stent occlusion and migration usually occur during the early phase after stent placement [37, 38]. Fortunately, no migration was noted within the duration of stent application in the present study; however, three patients presented with severe complications after stent occlusion. One patient presented with a pancreatic abscess, while another presented with a colon fistula, which was treated by observation. In addition, there was one case of splenic abscess, which was treated by percutaneous drainage. All three patients presented with diffuse multiple stones in the tail of the MPD. In our experience, the immediate complications of endoscopic stenting were mild, transient, and easily managed.

Statistical results of the present retrospective study revealed that “remaining stones during stent treatment” was the main factor for restenosis. There may also have been residual stones in the branch ducts in spite of cleaning the MPD during the stone retrieval treatments [35]. As many rates of diffuse stones were included in this study, the presence of stones in the side branches of the MPD must be taken into consideration after stent removal for long-term results.

In contrast to the study by Talamini et al., other studies including the present one found that neither resumption of alcohol consumption nor smoking after stent removal was associated with a significant increase in the rate of MPD restenosis [39]. Thus, the influence of tobacco use and alcohol consumption on MPD restenting outcome is still open to debate [5, 6, 39].

Despite the nearly statistically significant (*p* = 0.08) association between resumption of alcohol consumption after stent removal and MPD restenosis, a potentially important observation in this study is that alcohol prohibition should be continued not only throughout the duration of stent application but afterwards as well. Only two patients (4.9%) were able to abstain from smoking in this study. In future, we intend to evaluate the outcomes of MPD restenosis during smoking abstinence.

Importantly, the possibility of comorbid pancreatic cancer must also be considered during long-term EPS follow-up. Whereas most pancreatic duct strictures that occur during chronic pancreatitis are benign, a suspicion of malignancy requires prompt action involving surgical treatment rather than endoscopic stenting. All malignant cases were diagnosed by pancreatic brushing cytology in this study. Interestingly, MPD re-stricture did not aid in suspecting cases of malignancy; it was difficult to detect the presence of malignancy in two patients using imaging techniques such as enhanced CT and MRCP. Instead, the condition was diagnosed by routine pancreatic duct cytology. Previous studies have reported difficulties in diagnosing pancreatic malignancies arising in preexisting chronic pancreatitis [40, 41]. These facts indicate that in addition to cautious imaging follow-up, routine cytology must be performed after the treatment procedures.

The appropriate diameter as well as the duration of placement of the stents have not been determined in the present study. The use of the 10 Fr S-type plastic PS, which was replaced every 3 months, proved to be beneficial for the patients in this study; hence, this could be considered as the first line of treatment for both short-term and long-term endoscopic pancreatic stenting.

However, in this study, we experienced a serious complication concerning stent occlusion due to the presence of diffuse stones that remain in the tail of the MPD. Therefore, alternative methods such as multiple plastic stents and self-expandable covered metallic stents, as well as other surgical treatments, should also be thoroughly discussed for the treatment of refractory MPD strictures [42–46]. Further extensive studies involving pancreatic stents are required in future. In long-term stent application, it is important not to continue with the placement of an endoscopic stent in refractory cases in order to prevent pancreatic dysfunction and the development of pancreatic cancer. Therefore, it is important not to stick to the endoscopic stent placement in refractory cases, recurring pancreatitis exacerbation, and long-term stent application.

The current study is associated with some limitations. Since it is a study in a few cases (small sample size), there are some limitations in referring in this discussion. This was a retrospective and single-center study and limited external validity to this study; therefore, the possibility of unintentional selection bias cannot be fully excluded. Multivariate analysis data for risk of MPD restenosis (OR and 95% CI) was wide, and risk factors of pancreas cancer were not assessed in this study. This might have affected the outcome of small samples, so the results of this analysis cannot be generalized to other geographical regions of the world.

Despite this limitation, some factors indicated the statistical significance of the outcomes. Our explanatory analysis proceeded the use of 10 Fr S-type plastic pancreatic stents with routine exchange or both short-term and long-term outcomes in chronic pancreatitis patients with benign pancreatic duct strictures and impacted pancreatic stones, and this research is thought to lead to the next study. Therefore, our findings need to be confirmed in a prospective study.

In conclusion, we herein demonstrate that using 10 Fr S-type plastic PS with routine exchange is effective for both short-term and long-term outcomes. It is effective and useful in chronic pancreatitis patients with benign pancreatic duct strictures and impacted pancreatic stones.

## Figures and Tables

**Table 1:** Patient characteristics.

	*N*
Gender, male/female	47/12
Age, median (ranges)	56 (25–81)
Etiology	
Alcoholic (%)	51 (86.4)
Not alcoholic (%)	8 (13.6)
(Idiopathic/iatrogenic)	(7/1)
Stricture location	
Head/body/head + body/Santorini duct	48/6/3/2
Pancreatic stone location	
Single/diffuse	16/43
Smoke, yes/no	53/6

**Table 2:** Short-term outcomes: during EPS placement.

	Success	Failure	*p* value
Results (%)	41 (69.5)	18 (30.5)	
Stent placement period, median	276	—	
(ranges)	(30–589)	—	
Exchanges, total	169	—	
No. of exchange, median (ranges)	4 (1–16)		
EPS placement; Santorini duct/Wirsung duct	3/38	—	
No. of times of ERCP until the 10 Fr EPS placement, median	3.5	—	
^1^Pain relief (%)	37 (90.2)	15 (83.3)	0.19
Additional treatment			
None	11	4	
Surgery	0	4	
ESWL	30	10	
Reasons for failure			
Lithotripsy failure (ESWL, EHL)	—	8	
Deep cannulation failure	—	10	
^1^Stone location			
Single stone/multiple stones	12/29	5/13	0.62
^1^Stone extraction results (%)	37 (90.2)	4 (0.22)	<0.01^2^
EPST/precut	37/4	3/15	
PD dilation procedure device			
SSR	24 (58.5)	0	
SBDC	14 (34.1)	1	
EPDBD	3 (7.3)	17	
Complications			
Abdominal pain after stent placement	3	0	
Stent occlusion (complications pancreatitis/pancreatic abscess/colon-fistula/splenic abscess)	4 (1/1/1/1)	0	
Dislocation			
EPST hemorrhage	1	0	
Pancreatitis	3	1	
(Post-ERCP/post-ESWL/post-EHL)	2	3	
GW perforation	(0/1/1)	(1/0/2)	
Pseudocyst rupture	1	3	
	0	1	

^1^ *p* values: chi-square test. ^2^Statistically significant. SSR: Soehendra stent retriever catheter; SBDC: Soehendra biliary balloon dilator; EPDBD: endoscopic pancreatic duct balloon dilation.

**Table 3:** Long-term outcomes after stent removal.

Events	EPS success	EPS failure	*p* value
*N*	41	18	—
^1^Follow-up periods (month, median)	26.0	36.0	0.20
Location of stricture			
Head/body/head + body/dorsal-duct	12/1/2/2	15/3/0/0	
MPD restenosis (%)	17 (41.5)	—	—
^2^Time to restenosis (days, median)	191		
Causes of restenosis			
Remaining stones (%)	7 (17.1)		
Resumption of alcohol (%)	4 (9.8)		
Major papilla restenosis (%)	3 (7.3)		
Recurrence of stones (%)	2 (4.9)		
Restenting (%)	16 (39.0)	—	
Re-placement period (days, median)	260		
Complications			
Pancreatic abscess	1 (36)	0	
Papillary restenosis	1 (359)	0	
Liver abscess	1 (37)	0	
^3^Coexisting malignant disease (%)	3 (5.9)	1 (2.9)	0.64
Pancreatic cancer (%)	3 (5.9)	0	
(Diagnosed day after 1st EPST, median)	(211)	—	
Pharyngeal cancer (%)	0	1 (2.9)	
(Diagnosed day after 1^st^ EPST, median)	—	(1613)	

^1^ *p* values: Mann-Whitney *U* test. The following month was counted after the first performance of EPST. ^2^Counted from the EPS removal day when MPD dilation effect was revealed. ^3^*p* values: chi-square test.

**Table 4:** Risk factors for MPD restenosis (univariate analysis).

	Restenosis		OR (95% CI)	*p*
	(+)	(−)		
^1^Alcohol etiology of chronic pancreatitis +/−	16/2	20/3	1.2 (0.18–8.07)	0.62
^1^Resumption of alcohol after stent removal +/−	4/13	1/23	7.07 (0.71–70.19)	0.08
^1^Continued smoke +/−	17/0	22/2	—	—
^1^Single/multiple stones	5/13	6/15	1.04 (0.26–4.21)	0.95
^1^Remaining stones after stent removal +/−	6/12	1/22	11.1 (1.18–102.38)	^**2**^ **0.02**
^1^Recurrence of stones during stenting treatment +/−	3/14	0/22	—	—
^1^Stricture at the body of MPD +/−	5/12	1/21	0.11 (0.01–1.09)	^**2**^ **0.04**

^1^Unordered categorical variables. ^2^Statistically significant.

**Table 5:** Risk factors for MPD restenosis (multivariate).

	Restenosis		OR (95% CI)	*p*
	(+)	(−)		
^1^Remaining stones after stent removal +/−	6/12	1/22	11.44 (1.22–107.4)	^**2**^ **0.03**
^1^Associated body of MPD strictures +/−	5/12	1/21	0.17 (0.02–1.88)	0.14

^1^Unordered categorical variables. ^2^Statistically significant.

**Table 6:** Risk factors for pancreatic cancer (univariate analysis).

	Coexist cancer		OR (95% CI)	*p*
	(+)	(−)		
Alcohol etiology of chronic pancreatitis +/−	3/0	33/5	—	—
Resumption of alcohol after stent removal +/−	0/3	3/35	—	—
Continued smoking +/−	3/0	35/3	—	—
Single/multiple stones	2/1	26/10	0.77 (0.06–9.45)	0.84
Remaining stones after stent removal +/−	0/3	31/7	—	—
Re-stricture during stenting treatment +/−	0/3	17/21	—	—
Re-stricture with diffuse pancreatic stone +/−	0/3	12/26	—	—
Re-stricture, diffuse stone with an alcohol etiology +/−	0/3	12/26	—	—

## Data Availability

The data that support the findings of this study are available from the corresponding author (Ito K) upon reasonable request.

## Additional Points

*Core Tips*. 10 Fr S-type plastic pancreatic stents are effective for the treatment of pancreatic duct strictures and pancreatic stones in chronic pancreatitis.
